# Lumbar Scoliosis Combined Lumbar Spinal Stenosis and Herniation Diagnosed Patient Was Treated with “U” Route Transforaminal Percutaneous Endoscopic Lumbar Discectomy

**DOI:** 10.1155/2017/7439016

**Published:** 2017-01-19

**Authors:** Binbin Wu, Shaobo Zhang, Qingquan Lian, Haibo Yan, Xianfa Lin, Gonghao Zhan

**Affiliations:** ^1^Department of Anesthesiology and Pain Medicine, The Second Affiliated Hospital and Yuying Children's Hospital of Wenzhou Medical University, Wenzhou 325027, China; ^2^Department of Anesthesiology and Pain Medicine, The Hospital of Integrated Traditional and Western Medicine, Taizhou 317500, China; ^3^Department of Orthopaedics, The First People's Hospital of Wenling, Taizhou 317500, China; ^4^Department of Anesthesiology and Pain Medicine, The First People's Hospital of Wenling, Taizhou 317500, China

## Abstract

The objective was to report a case of a 63-year-old man with a history of low back pain (LBP) and left leg pain for 2 years, and the symptom became more serious in the past 5 months. The patient was diagnosed with lumbar scoliosis combined with lumbar spinal stenosis (LSS) and lumbar disc herniation (LDH) at the level of L4-5 that was confirmed using Computerized Topography and Magnetic Resonance Imaging. The surgical team preformed a novel technique, “U” route transforaminal percutaneous endoscopic lumbar discectomy (PELD), which led to substantial, long-term success in reduction of pain intensity and disability. After removing the osteophyte mass posterior to the thecal sac at L4-5, the working channel direction was changed to the gap between posterior longitudinal ligament and thecal sac, and we also removed the herniation and osteophyte at L3-4 with “U” route PELD. The patient's symptoms were improved immediately after the surgical intervention; low back pain intensity decreased from preoperative 9 to postoperative 2 on a visual analog scale (VAS) recorded at 1 month postoperatively. The success of the intervention suggests that “U” route PELD may be a feasible alternative to treat lumbar scoliosis with LSS and LDH patients.

## 1. Introduction

Lumbar spinal stenosis (LSS) is the most common spinal degenerative condition and usually related to the occurrence of low back pain (LBP), functional limitations, and disability [1]. The causes can be intervertebral joint hypertrophy, osteophytes, and lumbar disc herniation (LDH) [2]. It has been reported that almost 9% of general population and about 47% of people older than 60 years are diagnosed with LSS and their 2-year cost of treatment is 4 billion dollars in the United States alone. LSS is one of the most common spinal pathologies affecting patients that are older than 65 years [3, 4]. In addition, approximately 80% of Chinese adults with LSS experience low back or leg pain or both during their lifetime [5]. Majority of patients have significant pain alleviation through massage and physical therapies, but approximately 20% suffer from intractable pain and suffer greatly [6].

Open discectomy (OD) has been regarded as the standard surgical procedure for LSS during the last decades [7]; however, OD needs to extensively resect the lamina in the regions of facets, causing iatrogenic instability and more postoperative morbidity [8], such that the outcome is not satisfying [9]. Recent advancements in minimal invasive discectomy operations include the transforaminal percutaneous endoscopic lumbar discectomy (PELD) approach that has many advantages compared to older techniques in terms of protecting the lamina, muscles, ligaments, and spinal canal, as well as long-term success by minimizing postoperative pain, epidural scarring, segment instability, and slippage [7, 8, 10]. Despite the advantages, the applications of “U” route PELD are limited due to controversy regarding its therapeutic efficacy and indication to treat LSS and LDH [11].

The objective of the case report was to describe the “U” route PELD technique, which could effectively treat lumbar scoliosis combined with lumbar stenosis, caused by herniation and/or osteophyte on L3-4 and L4-5 discs, with the aim of enriching the knowledge and further applications of “U” route PELD.

## 2. Case Presentation

### 2.1. History and Examination

A 63-year-old man presented with LBP and left leg pain for 2 years with symptoms worsening in the recent 5 months. The patient consulted our department for treatment, and the neurological examination revealed lumbar scoliosis, limited lumbar spine flexibility, L3-4 interspinous tenderness, and marked tenderness on the left side of L5. In addition, the patient reports radiating pain on the left leg and weakened shallow feel on both lower limbs. The straight leg raising test on the two sides and pelvic compression test were negative, and no muscle weakness or reflex was found. The visual analog scale (VAS) pain ratings of LBP and left leg pain were both reported as 9 of 10.

**Figure 1:**
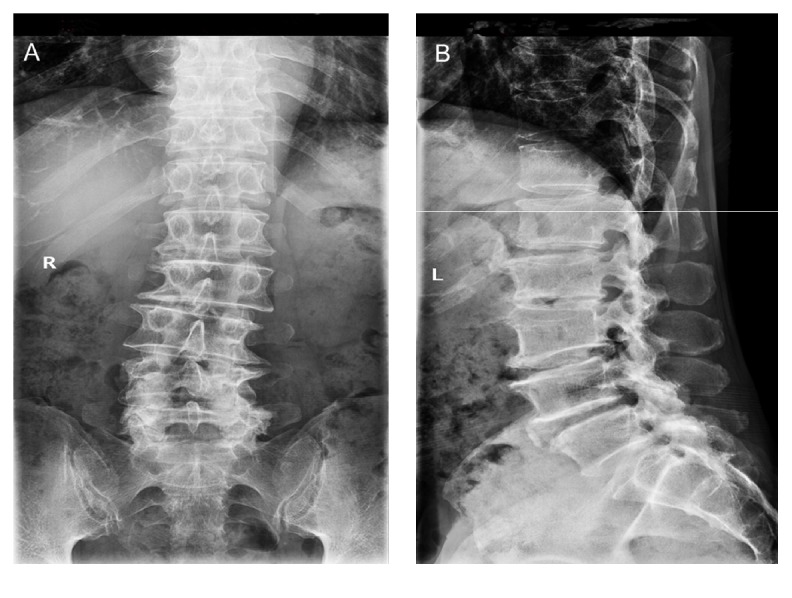
The preoperative anteroposterior (A) and left lateral (B) X-ray images of the patient, showing lumbar scoliosis, lumbar degeneration, and vertebral instability.

**Figure 2:**
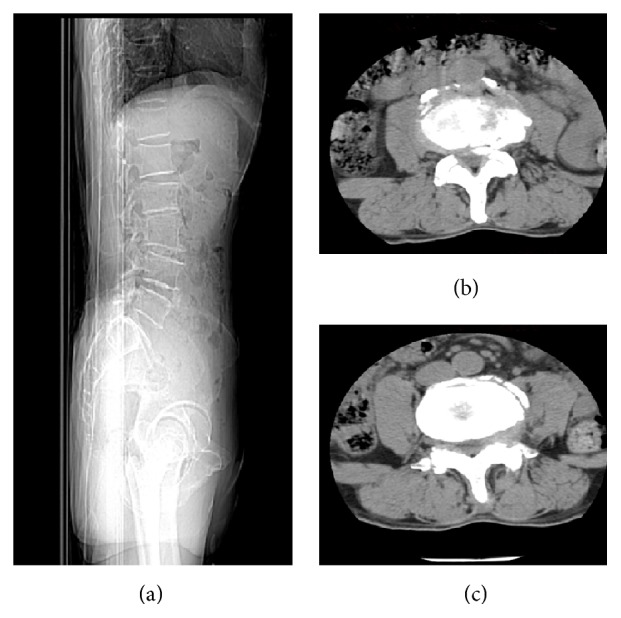
The sagittal (a) and coronal CT images of L3-4 (b) and L4-5 (c) revealed lumbar stenosis combined with intervertebral disc herniation at both levels and combined lumbar facet joint degenerative changes.

**Figure 3:**
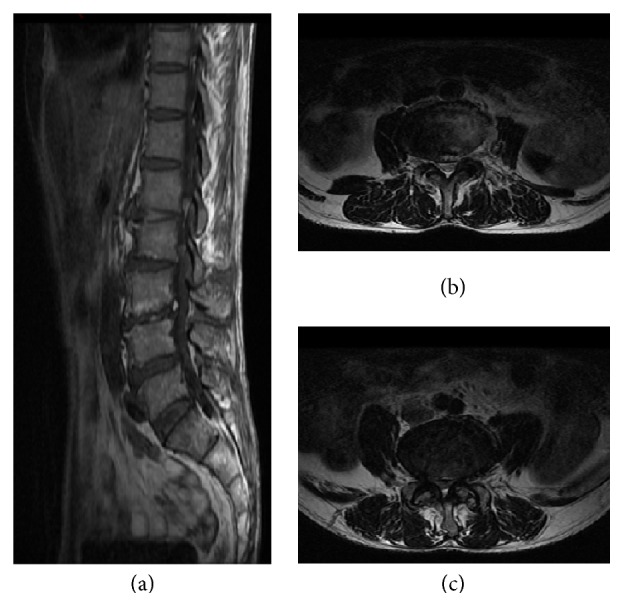
Both the T1 sagittal (a) and coronal MRI images of L3-4 (b) and L4-5 (c) suggested that the herniation and lumbar stenosis compressed the thecal sac at both levels, and lumbar degenerative changes were also reported.

The lumbar X-ray examination revealed lumbar scoliosis, lumbar degeneration, and L2 vertebral slip (Figure 1). The Computerized Topography (CT) indicated a L3-4 and L4-5 lumbar stenosis combined with intervertebral disc herniation and lumbar joint facets degeneration unexpected for his age (Figure 2). The Magnetic Resonance Imaging (MRI) confirmed results from the CT scan but also suggested lumbar stenosis at L3-4 and L4-5; thecal sacs at the both levels were compressed by herniation (Figure 3). After completion of preoperative tests and examinations, we estimated that the existing lumbar stenosis at L3-4 and L4-5 was due to spinal osteoarthritis and herniation and that of L4-5 was more serious. Meanwhile, based on the patient's clinical history, we concluded that conservative physical treatments might be ineffective and a spine surgery would be a better choice. After the patient was informed of the disadvantages and advantages of both OD and “U” route PELD, he chose “U” route PELD surgery.

### 2.2. Intervention

**Figure 4:**
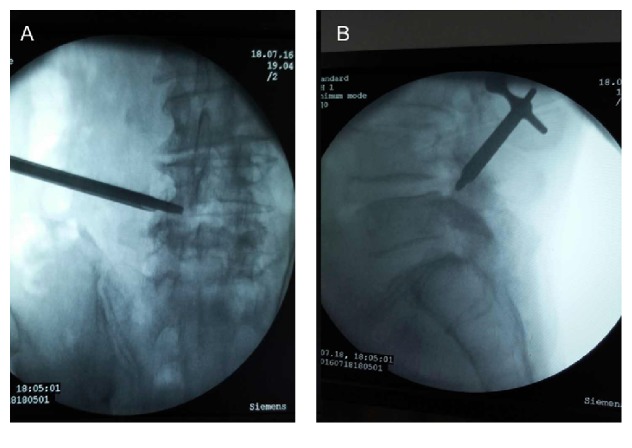
The location pictures after successfully reaching the operation area at L4-5 with a guide bar.

All procedures were performed following the standard transforaminal endoscopic discectomy technique after local anesthesia was administered [12]. The patient lay prone on an operating table on the contralateral side, and C-arm fluoroscopy technique was used to determine the affected discs and pedicles. Thus, the surgeons drew lines from the mid-pedicular annulus of L3-4 and L4-5 to the facet lateral margin and extended them to the body surface, and the skin entry point was about 10 cm from the midline. After a routine disinfection procedure, subcutaneous tissue and trajectory tract were infiltrated with 1.0–1.5 mL of 1% lidocaine at the L4-5 level. Following this, an 18-gauge needle was inserted to reach the facet of L5 superior articular process under a fluoroscopic guidance, and with a puncture angle of about 15°. Then, we retreated the stylet followed by injecting another 20 mL 1% lidocaine for further anesthesia and inserted a guide wire as the direction of the needle. After that, the needle was retreated and a 0.8 cm incision was made at the position of guide wire firstly; secondly, a serial dilation and working channel were inserted as the direction of guide wire; thirdly, we retreated the guide wire and dilation and inserted the guide bar into the working channel. To prevent the occurrence of postoperative spinal instability, the guide bar was passed over the facet of L5 superior articular process without damaging any bone tissue. However, at that moment, the patient complained radiating pain on his left leg when the surgeon planned to insert the guide bar into his spinal canal. We estimated that the pain resulted from nerve root compression as we repeatedly adjusted the position of the guide bar. However, all adjustments could not avoid touching nerve root and the pain was persistent. Therefore, we changed the puncture path to the superior and interior articular process facets at the level of L4-5 and resected the facets partly for decompression. After the guide bar being inserted into the posterior of thecal sac (Figure 4), the working channel was rotated around the direction of the guide bar, and the endoscope was introduced. Besides, a continuous irrigation system for a clear endoscopic view was used. Then, we removed the osteophyte and reshaped the ligamentum flavum firstly. Following that, the working channel was adjusted to remove the herniation mass in the gap between posterior longitudinal ligament and thecal sac, just like a “U” route. At the end, the operative field was copiously irrigated and meticulous hemostasis was obtained, and suture was placed at the incision after the channel was removed.

**Figure 5:**
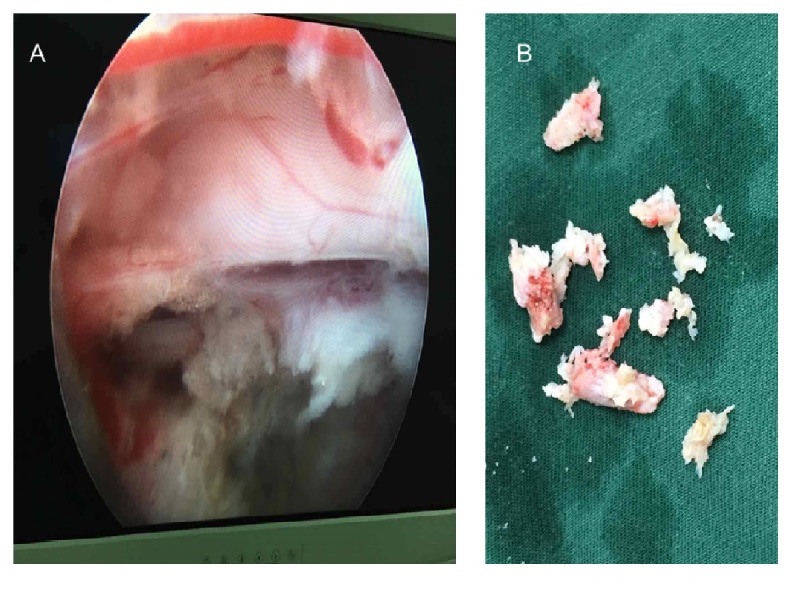
The sight under the endoscope at L4-5 (A); the herniation mass and bone hyperplasia were collected at the plate (B).

**Figure 6:**
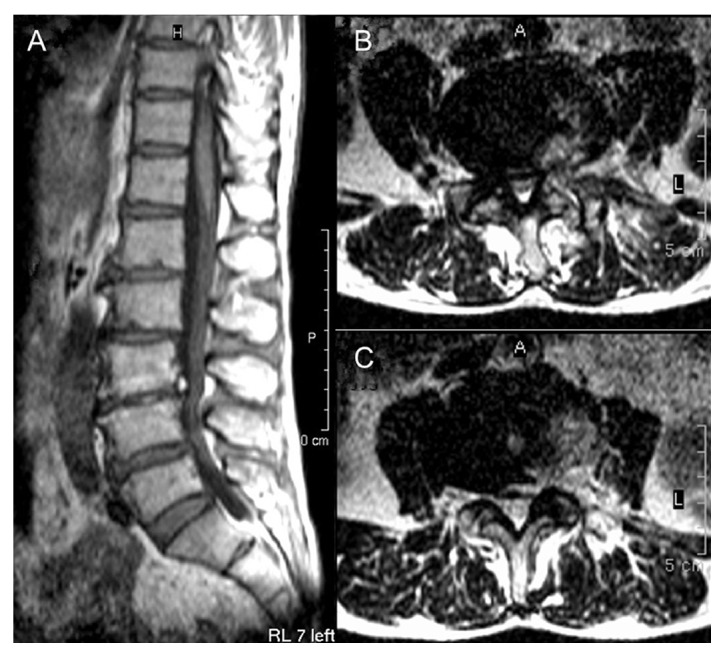
Both the T1 sagittal (A) and coronal MRI images of L3-4 (B) and L4-5 (C) at 1-month postoperatively, indicating that the herniation and stenosis at both levels were alleviated compared with the preoperative results, despite edema being observed.

The spinal stenosis was also observed at the L3-4 level; for further treatment, we inserted the guide bar to reach the location of L4 superior articular process facet after the determination of landmarks and skin window as described above. After local anesthesia, the working channel reached the location of herniation and osteophyte at L3-4 as the guidance of the guide bar, and the mass between thecal sac and posterior longitudinal ligament was removed under endoscope successfully. At the end of the operation, the patient reported the low back pain was alleviated, the VAS pain rating was about 2 of 10, and the leg pain absolutely disappeared. All of the resected mass was collected on a plate (Figure 5), and the irrigation, meticulous hemostasis, and suture were done as described above. Thus, treating the LSS mainly caused by LDH and osteophyte combined lumbar scoliosis with PELD was performed. An MRI scan was done 1 month postoperatively; in addition, the patient reported his LBP 1 of 10 on a VAS scale and 0 of 10 on a VAS scale of leg pain. MRI imaging 1 month postoperatively suggested disc edema at L3-4 and L4-5; herniation and stenosis were alleviated compared with preoperative images (Figure 6).

## 3. Discussion

It has been demonstrated that PELD is a relatively safe, minimally invasive procedure for LSS and LDH compared with OD, with merits such as less tissue trauma and blood loss, shorter mean disability period, and recovery time. The procedure requires a small incision of 0.6–0.8 cm and 3 days of in-patient stay [7, 13, 14]. Despite the above advantages and inspiring clinical results, PELD is not universally adopted because of some disadvantages, such as difficulty in anatomical delineation during the endoscopic approach and the learning curve to disassociate the neural structure from the instruments or to develop skillset and experience to safely perform the surgery. In order to circumvent iatrogenic persistence of neuropathic postoperative pain, many new techniques have been developed, and the PELD is also being advanced [15–20]. Several years ago, PELD was not a recommended therapy for patients with highly migrated herniation and lumbar stenosis, but a recently developed “U” route PELD becomes an available treatment for these pathologies regardless of laterality or herniation [8, 21]. And we can reach the operation area with the working channel bypassing the vertebral facets without destroying any anatomical structure of the spine. But for this case, because of inducing radiating pain when we rotated in guide bar, we resected the articular process facets at L4-5 for decompression for this patient. Lumbar scoliosis leads to mispositioning during the surgery, and we circumvented this potential issue by adjusting the direction and orientation of the needle repeatedly, although the needed may be stopped several times due to anatomical channel abnormalities in the process. As a result, an osteophyte formation with majority developing near the ligamentum flavum was treated, and then we changed the direction of working channel to the gap between posterior longitudinal ligament and thecal sac; this is just like a “U” route, a newly developed approach of PELD, and is heatedly discussed; however, there are not many studies published about it. And during a 3-month follow-up, no spine instability was observed as a result from the surgery. Moreover, many studies have suggested endoscopic disc surgery by experienced and well-trained surgeons can achieve more favorable and sustainable clinical results equivalent to the standard microsurgical technique [20, 22]. Therefore, the clinical outcome of PELD is closely related to the proficiency of surgeons. The surgeon of the operation in this study has carried out more than 2,000 cases of LBP with PELD, including LSS and LDH patients, in China alone.

The patient's VAS pain rating decreased to 2 after the surgery and was 1 when he was discharged, no pain on his leg. And we cannot absolutely exclude the possibility that the slight pain at back may be related to minor injury of the paraspinal muscles during the operation, although the postoperative 1-month MRI indicated disc edema of both levels of L3-4 and L4-5. In addition, both the follow-up results at 1 month and 3 months after surgery suggested that no complications happened to the patient, despite the fact that there are no images for postoperative 3 months here. All these indicated the success of the surgery. With this case, we might demonstrate that the “U” route PELD could be an alternative treatment for patients diagnosed by lumbar scoliosis with LSS and LDH, but we also need a larger-sample study with long-term follow-up in this area.
